# Effects of fruit and vegetable intake on memory and attention: a systematic review of randomized controlled trials

**DOI:** 10.1186/s13643-024-02547-8

**Published:** 2024-06-07

**Authors:** Khai Ling Khor, Vashnarekha Kumarasuriar, Kok Wei Tan, Pei Boon Ooi, Yook-Chin Chia

**Affiliations:** 1 Department of Medical Sciences, School of Medical and Life Sciences, Sunway University, Selangor, Malaysia; 2 School of Psychology, DISTED College, Penang, Malaysia; 3 School of Psychology and Clinical Language Sciences, University of Reading Malaysia, Iskandar Puteri, Malaysia; 4 Department of Primary Care Medicine, Faculty of Medicine, University of Malaya, Kuala Lumpur, Malaysia

**Keywords:** Fruits, Vegetables, Memory, Attention, Randomized controlled trial, Systematic review

## Abstract

**Background:**

Memory and attention are important for daily functioning, and their function deteriorates due to aging. However, fruit and vegetable consumption are one of the protective factors against deterioration in memory and attention. This systematic review of randomized controlled trials (RCTs) aims to identify the effects of fruit and vegetable consumption on memory and attention.

**Methods:**

We conducted a systematic search in EBSCOhost, ProQuest, PubMed, Embase, and Web of Science from inception up to 06/09/2022. The inclusion criteria were peer-reviewed articles, fruit and vegetable intake measured using randomized controlled trials, and the outcome measures that showed the results of memory and attention scores. Two researchers independently extracted articles that met the selection criteria and evaluated the quality of each study.

**Results:**

There were 70 articles identified from the databases, of which 13 articles met the inclusion criteria and were included in this systematic review. There were 493 participants in total. The results show that consumption of fruit and vegetable intake improved memory and attention in longitudinal studies (10 to 12 weeks). Children showed improvement in immediate recall after supplementation with blueberries. Older adults required a higher dose of fruit and vegetable intake consumption to achieve significant improvement compared with children and younger adults. Furthermore, the effect of fruits and vegetables on memory showed better immediate memory recall than delayed recall.

**Conclusion:**

This systematic review showed that there is an improvement in memory and attention with fruit and vegetable intake consumption. Hence, awareness of fruit and vegetable intake consumption is important to maintain cognitive health.

**Supplementary Information:**

The online version contains supplementary material available at 10.1186/s13643-024-02547-8.

## Introduction

Fruits and vegetables have numerous nutritional benefits, such as high concentrations of nutrients, including vitamins, minerals, fibre, carotenoids, flavonoids, and phytochemicals [1–3]. Phytochemicals play a nutraceutical role [4] and function as antioxidants and scavengers for free radicals that have been shown to slow cognitive deterioration [5]. Fruits such as blueberries, cherries, mulberries, grapes, and *Lycium barbarum* (goji berries) contain dietary polyphenols [6–9] and phytochemicals [10], which are associated with delay of the onset of cognitive decline [11].

Cognitive decline heralds dementia, which is a global issue now where the World Health Organization (WHO) has projected that 152 million people will have dementia in 2050 [12]. Dementia symptoms include gradual, progressive memory loss and attention deficits [13]. Individuals who lose attention have difficulty learning new things and trouble reading long texts. Furthermore, individuals (studies including patients only or including nonpatients as well) with memory loss, such as episodic memory loss, tended to forget daily events includes taking medicine or appointments. In summary, cognitive decline affects an individual’s daily functioning [14, 15] and workplace performance [16]. One of the reasons for memory deterioration is brain aging, especially oxidative stress in the hippocampus [17, 18], as the hippocampus stores long-term memory [19]. In addition to memory loss, dementia patients also suffer from attention deficits. Attention involves various anatomical areas, including the thalamus and the occipital, temporal, parietal, and frontal cortices, which form a neural network [20]. The attention neural network is influenced by the rates of neuron firing [21, 22], and unfortunately, demyelination occurs due to aging, which slows information processing [23, 24] and affects attention.

**Fig. 1:**
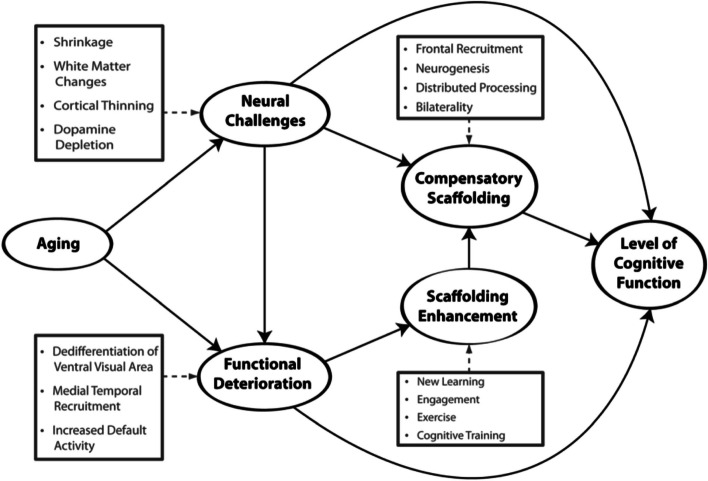
Conceptual model of the Scaffolding Theory of Aging and Cognition (STAC) [25]

The theoretical framework guiding this study is the Scaffolding Theory of Aging and Cognition (STAC) [25, 26]. STAC is a conceptual model that integrates biological aging and environmental factors that affect cognitive function and the dynamic interaction with protective factors and compensatory process in the brain. The theory suggests that as we age, our brains develop compensatory mechanisms, which are referred to as scaffolds, to maintain cognitive function, countering the effects physiological aging [25, 27] (refer to Fig. 1). One of the compensatory mechanisms is lifestyle choices, such as dietary intake. Previous studies have shown that an adequate amount of fruit and vegetable intake is an effective preventive measure against cognitive decline [28–30]. Fruits and vegetables are rich in neuroprotective compounds and may act as cognitive scaffolds in the context of STAC.

Various forms of fruit and vegetable intake have been explored in previous studies, for example, in the form of capsules [31, 32], juices [33–38], powder [39–41], and frozen fruits [42]. Additionally, the efficacy of cognitive function was also measured by different cognitive tests. For example, memory is measured by the California Verbal Learning Test (CVLT) [35], the Computerized Mental Performance Assessment System (COMPASS) [33], the Visual Verbal Learning Test (VVLT) [36], the Spatial Paired Associate Learning Test [35], and the Visual Spatial Learning Test [36]. Furthermore, attention is also measured by different methods, e.g. the Stroop test [31, 37, 40, 43], Frankfurt Attention Inventory [32], Trail Making Test [34, 38], Auditory Odd Ball [40], Modified Attention Network Task [42], Auditory Continuous Performance Test [32], and COMPASS [33].

From the various forms of fruits and vegetables and cognitive tests, research has shown inconsistent results. While some studies showed a significant impact of the effectiveness of fruits and vegetables on attention [31, 32, 38], other studies showed no significant result [37, 40, 42, 43]. In addition, the studies that measured the efficacy of memory also showed mixed results, whereby the studies from several studies [34–36, 39, 42] showed a significant result, whereas studies from others did not [33, 37, 38]. Because of the conflicting results in studies, this systematic review aims to synthesis existing research of the effects of fruit and vegetable intake on memory and attention in randomized controlled trials in different age groups.

## Materials and methods

**Fig. 2:**
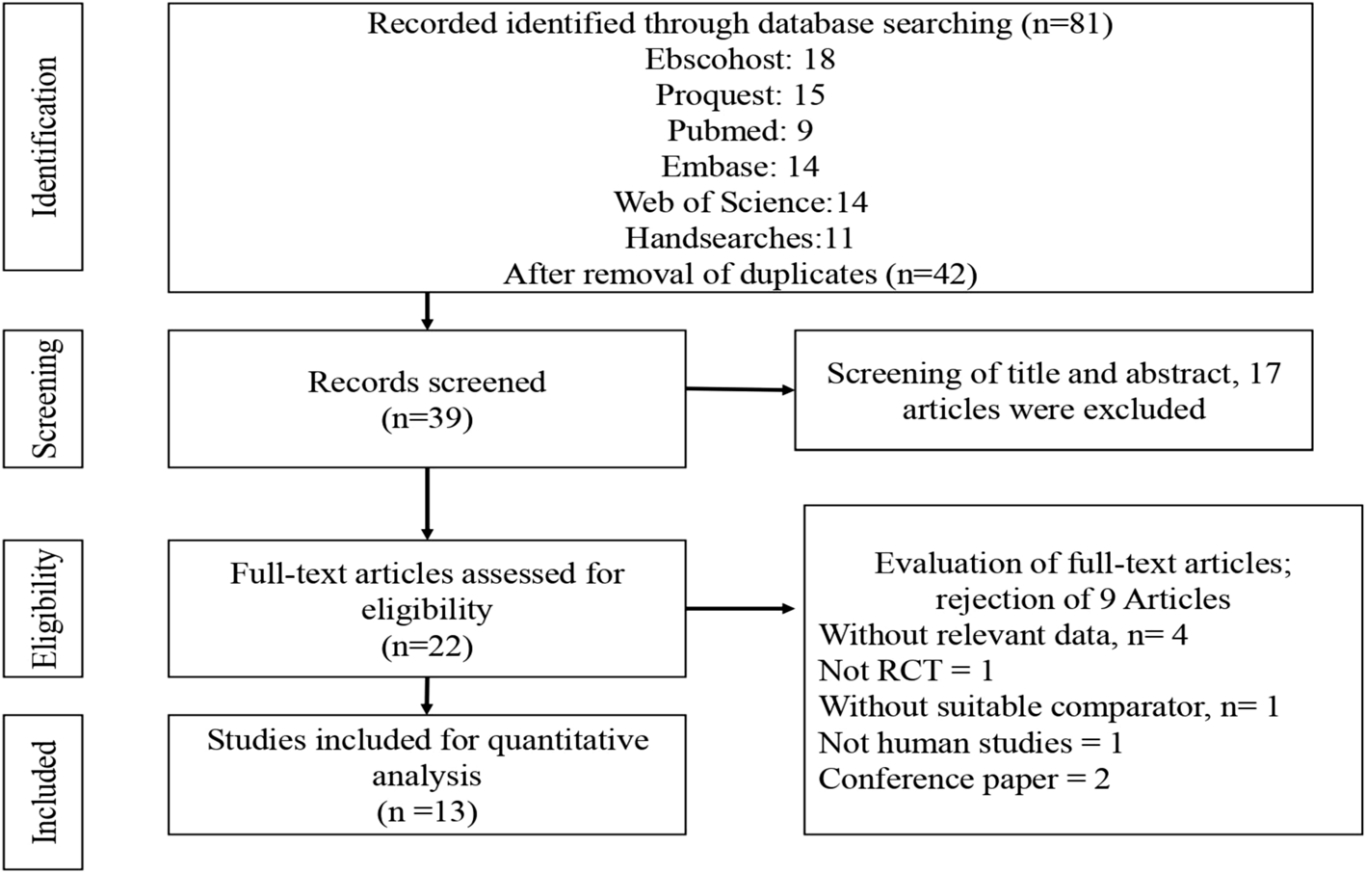
Prima Flowchart

This study was registered at PROSPERO (ID: CRD42022308658). It was conducted according to the Preferred Reporting Items for Systematic Reviews and Meta-analyses (PRISMA) guidelines (refer to Fig. 2). This study only involved secondary data retrieval and analysis, so no ethical approval was needed or sought.

### Literature search

Two investigators (KKL and VK) independently searched EBSCOhost, ProQuest, PubMed, and Web of Science on 06/09/2022. The following search terms were used: (fruit OR vegetable OR plant capsule) AND (selective attention OR divided attention OR sustained attention OR attention) AND (memory OR sensory memory OR short-term memory OR long-term memory OR working memory OR episodic memory OR semantic memory OR procedural memory OR autobiographical memory OR iconic memory OR echoic memory OR semantic memory OR declarative memory) AND (intervention OR randomized OR RCT or placebo* OR clinical trial) (refer to Appendix 1).

All searches were conducted by EndNote 20, and duplicated articles were removed. Then, titles and abstracts were screened to identify relevant articles to be included. Finally, articles with full texts were assessed for eligibility in this systematic review.

### Inclusion criteria and exclusion criteria

Any experimental studies that reported the consumption of any types of fruits and vegetables with the outcome measures of memory and attention that fulfilled the inclusion criteria were analysed. The inclusion criteria were peer-reviewed articles; randomized controlled trials; studies that included any fruits or vegetables either in the form of capsules, powder, or fresh fruits or vegetables; and outcome measures that were memory and attention scores. The exclusion criteria were conference papers, proposals, and outcome measures that did not have the mean score for pre- and post-intervention.

### Study selection

All relevant articles meeting the inclusion criteria were imported into Endnote software, version 20. Identifying duplication was performed first. In the study selection process, two investigators (KKL and VK) screened the titles and abstracts and then filtered out the full-text articles that did not meet the inclusion criteria. At this stage, disagreements primarily arose, because of uncertainties on the abstract as to whether it actually fulfilled our inclusion criteria. For example, articles involving Mediterranean diet were extracted during the selection phase. But these studies involving the Mediterranean diet emphasized not only plant-based foods such as fruits and vegetables but also on, nuts, whole grains, etc. Hence, during the review process, it became apparent that articles discussing special diet plans, which did not exclusively focus on fruits and vegetables, had to be excluded. To address these discrepancies, any disagreement between investigators was resolved through discussions with CYC, OPB and TKW before reaching a final decision.

### Data extraction

Data such as the last name of the first author, year of publication, country, objectives of the study, study design, intervention duration, sample size, gender, age, health status, and measures of the study were extracted from the articles. In addition, the outcome measures included data on memory and attention, with baseline means and standard deviation, final means and standard deviation, and the *p* value for the difference in mean change between the intervention and control groups. Two investigators (KKL and VK) extracted the data independently, and any differences were resolved between them. An example of a difference was the variation in the reporting of cognitive function. Specifically, some articles presented results as the number of correct answers, while others expressed it as the percentage of mean changes. To address these discrepancies, a thorough discussion was conducted and the final decision was based on a consensus between the two investigators.

### Quality assessment

The two investigators (KKL and VK) used the Revised Cochrane risk-of-bias tool for randomized trials (RoB2) to assess the risk of bias for each study independently [44]. All sources of bias (randomization process, deviation from intended intervention, missing outcome data, measurement of the outcome, selection of the report result) were evaluated accordingly. Any differences were discussed and resolved by the investigators.

## Results

### Description of included studies

The literature search and selection processes are presented in Fig. 2 in the appendix. From the literature search, 81 articles were identified from databases in the initial screening. After removing 42 duplicate articles, 39 articles were identified for review of the title and abstract. After screening the title and abstract, 17 articles (such as studies focusing on medicinal plants or review papers) did not fulfil the objective of our study and were excluded. The remaining 22 articles that fulfilled our criteria were further assessed by reading the full text of the articles. With the evaluation of full-text articles, only 13 articles were eligible for quantitative analysis, while nine were excluded because the studies did not include relevant data, such as pretest results (four articles), was not a randomized controlled trial (one article), without a suitable comparator (one article), not a human study (one article), or conference papers (two articles).

### Characteristics of included studies

Thirteen [13] articles were finally included in this systematic review (Table 1). As these articles used different memory and attention measuring tools for their baseline and outcome measures, they are not suitable for meta-analysis.

### Quality assessment

**Fig. 3:**
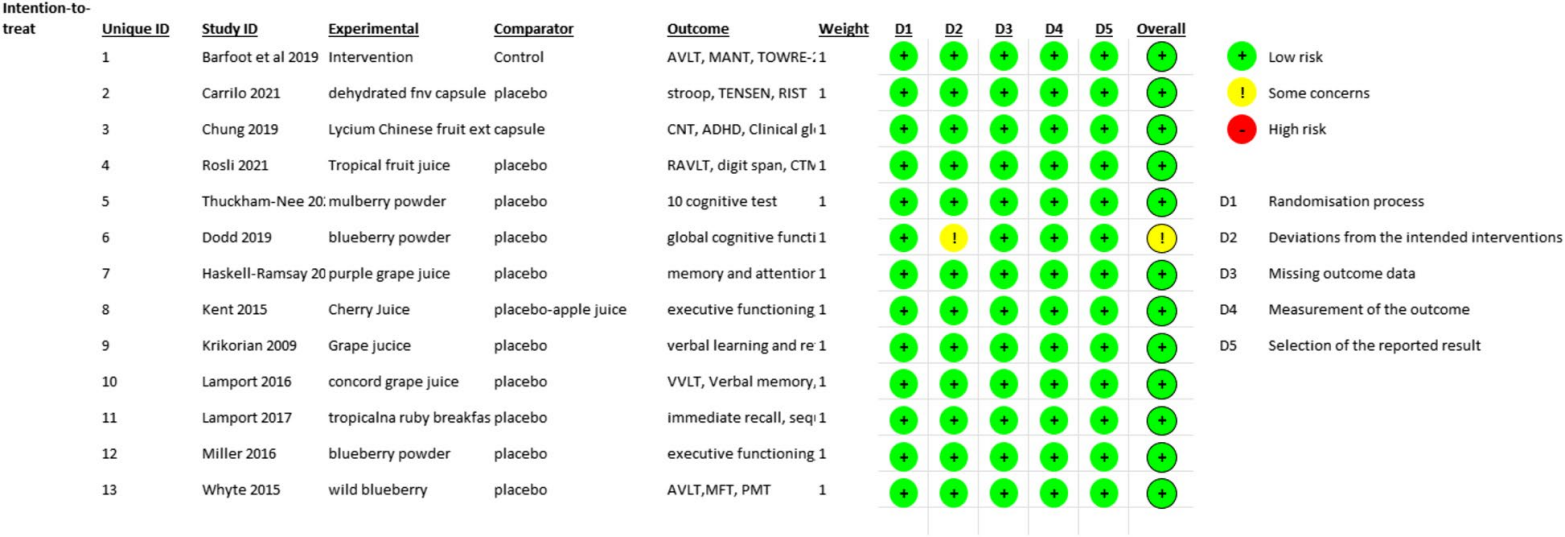
Assessment of risk of bias

The assessment of risk of bias is summarized in Fig. 3. Most of the studies have described the randomization process and allocation of intervention or placebo with sufficient details. Thus, they were judged as having a low risk of bias within this domain. Only one study had one concern—the study design was not clear if it was a single-blinded or double-blinded study [43]. However, the study was still included because it still provided data on the efficacy of fruit and vegetable intake consumption on attention and memory. Furthermore, this study was a crossover design where all the participants participated in all the arms.

### Participants

There was a total of 472 participants from the 13 articles, with 179 (37.9%) males and 293 (62.1%) females. The ages of all participants ranged from 6 to 80 years. Nine studies were on healthy participants [31, 33, 36, 37, 39–43], one study on participants with mild-moderate dementia [34], one article on participants with early memory decline [35], one article on participants with poor cognitive function assessed by Rey’s Auditory Verbal Learning Test [38], and one article that did not report the health condition of the participants [32] ( Table 1).

**Table 1:** Characteristics of the included studies

Author; country	Study design	Intervention (duration)	Sample size (gender male; female)	Age (mean; SD)	Health condition	Types of intervention	Control ingredient
(Carrillo et al., 2021) [31] Spain	Randomized, crossover, double-blind, sex-stratified, place-controlled	16 weeks	92 (M, 47; F, 45)	32.74 ± NR	Healthy	Dehydrated fruits and vegetable capsules	Placebo made of microcrystalline cellulose
(Chung et al., 2019) [32] Korea	Randomized, double-blind, placebo-controlled, crossover trial	11 weeks (4 weeks of intervention + 3 weeks of washout + 4 weeks of crossover)	43 (M, 14; F, 29)	19.25 ± 2.29	Not reported	Lycium Chinese fruit capsule	Starch capsule
(Rosli et al., 2021) [38] Malaysia	Randomized, placebo-controlled trial	10 weeks (3 days per week)	31 (M, 0; F, 31)	50.8 ± 3.7	Poor cognitive function tested by RAVLT	A mixture of pomegranate concentrates with guava and roselle extract	No juice or natural polyphenol was present, but the supplement looked and tasted like TP 3-in-1™ juice with the same energy content
(Thukham-Mee et al., 2020) [40] Thai	Randomized, placebo-controlled, crossover trial	Acute effect (3 h)	46 (M, 20; F, 26)	IG: M = 8.83; SD = 0.38 CG: M = 8.87; SD = 0.35	Healthy	Mulberry powder	Milk
(Barfoot et al., 2019) [42] NR	Randomized, single-blind, parallel-group trial	Acute effect (2–6 h post-consumption)	54 (M, 25; F, 29)	IG: M = 8.24; SD = 0.88 CG: M = 8.23; SD = 10.05	Healthy	Freeze-dried wild blueberry	Sugar-matched placebo (8.9 g fructose, 7.99 g glucose, 4 ml vitamin c) + + 170 ml of cold tap water + 30 ml of low-flavanoid fruit squash = 200 ml drink
(Dodd et al., 2019) NR [43]	Randomized, crossover, controlled trial	Acute effect (2 h)	18 (M, 8; F, 10)	68.7 ± 3.3	Healthy	Freeze-dried wild blueberry	Sugars + Vitamin x + 1 g of citric acid`
(Haskell-Ramsay et al., 2017) [33] Newcastle	Randomized, placebo-controlled, double-blind, counterbalance, crossover trial	Acute effect (20 min)	20 (M, 7; F, 13)	21.05 ± 0.89	Healthy	Welch purple grape juice	Welch white grape juice + blackcurrant flavour cordial + cold water
(Kent et al., 2015) [34] Australia	Randomized, placebo-controlled trial	12 weeks	49 (M, 25; F, 24)	IG: M = 78.9; SD = 5.2 CG: M = 80.6; SD = 6.6	Mild-moderate dementia	Cherry juice	Commercially prepared apple juice
(Krikorian et al., 2009) [35] NR	Randomized, placebo, controlled trial	12 weeks	12 (M, 8; F, 4)	78.2 ± 5.0	Early memory decline	Concord grape juice	Beverage tastes like grape juice, without any natural polyphenol, but have some carbohydrate and energy
(Lamport, Lawton, et al., 2016) [36] UK	Randomized, crossover design	12 weeks	25 (M, 0; F, 25)	42.8 ± 0.7	Healthy	Concord grape juice	Beverage tastes like grape juice, without any natural polyphenol, but have some carbohydrate and energy
(Lamport, Pal, et al., 2016) [37] UK	Acute, single-blind, randomized, crossover trial	Acute effect (2 h)	24 (M, 4; F, 20)	22 ± 2.2	Healthy	Tropicana Ruby Breakfast Juice	Cordial drink concentrated + mineral water
(Miller et al., 2017) [39] NR	Randomized, double-blind, placebo- controlled trial	90 days/approximately 13 weeks	37 (M, 12; F, 25)	IG: M = 67.8; SD = 4.6 CG: M = 67.3; SD = 4.8	Healthy	Blueberry powder	Not reported
(Whyte et al., 2015) [41] UK	Double-blind, crossover, placebo trial	Acute effect (6 h)	21 (M: 9; F:12)	8.7 ± 0.67	Healthy	Blueberry powder	Squash juice

*Note.* *RAVLT *Rey’s Auditory Verbal Learning Test

All 13 trials included Western or Asian participants. Six trials were conducted in Western countries such as Spain [31], Australia [34], and the UK [33, 36, 37, 41]; three trials were conducted in Asia such as Korea [32], Malaysia [38], and Thailand [40]; and four trials did not report the country of research [35, 39, 42, 43].

### Supplementation: forms of fruits and vegetables, duration, and outcomes

#### Forms of fruits and vegetables

Four major forms of fruit and vegetable intake were used in these 13 studies: powder form made from fruits and vegetable extract, capsule, frozen fruits, and juices made from fresh fruits/vegetables.

Different types of fruits were extracted and reproduced in powder form. For example, blueberry powder was used by Miller et al. [39], Whyte et al. [41] and Dodd et al. [43], and mulberry powder was used by Thukham-Mee [40]. Supplementation with blueberry powder showed a significant improvement in short-term memory in the research of Miller et al. [39] and Whyte et al. [41], but there were no significant changes in the research by Dodd and colleagues (2019). Furthermore, Miller et al., Whyte et al. [41], and Dodd et al. [43] showed that there was no significant improvement in long-term memory. On the other hand, mulberry powder supplementation by Thukham-Mee [40] showed no significant improvement in either attention or memory. These studies of fruit-derived powder had yielded mixed results on changes in cognitive function. However, it is noteworthy that the duration and dosage of these supplementation varied across the studies included in our systematic review. This variation in duration and dose may contribute to the differences in cognitive outcome. The specific details regarding the duration and dosage will be discussed in later section below.

In terms of the use of capsules, Chung et al. [32] extracted Lycium Chinese fruits into capsule form, and Carrillo et al. [31] distributed capsules with a mixture of dehydrated fruit and vegetable. Both studies showed a significant improvement in both attention and working memory.

Only one study conducted by Barfoot et al. [42] used freeze-dried blueberries and blended them with milk. However, there was no significant improvement in either attention or memory in this study.

Different types of juices were used such as cherry juice [34], grape juice [33, 35, 36], commercial juices with high flavonoid content [37], and mixed juices [38]. The research using juices had mixed results. Cherry juice showed significant improvement in short-term memory and long-term memory but no significant changes in attention and semantic memory [34].

Grape juice supplementation showed a significant improvement in long-term memory, short-term memory, and spatial memory [36]. However, the results on total memory recall were mixed, where Krikorian et al. [35]showed a significant improvement, but Haskell-Ramsay et al. [33] showed no significant improvement.

Commercial juice did not show any significant changes in attention and memory [37]. On the other hand, mixed juiced supplementation only showed significant changes in attention when measured by the Comprehensive Trail Making Test (CTMT) Trail 1, but not CTMT Trail 2 to 5 and CTMT composite index [38]. Trail 1 is where participants connect all the numbers orderly; trail 2 is where participants are requested to connect all numbers orderly with empty circles as distractors; trail 3 is where participants have to connect all numbers ordinarily with empty circles and patterned circles as distractors; trail 4 is to connect numbers orderly where the numbers are presented in Arabic or letters; and trail 5 is where the participant has to connect the number and letter A to L orderly in an alternating sequence, for example, 1-A-2-B-3-C [45]. This result suggests that mixed juice supplementation had a positive effect on attention only and may not extend to more complex tasks involving distractors or demonstrate cognitive flexibility.

### Duration

There were two different intervention durations: acute effects and short-term effects. Acute effects were defined as outcomes that were measured immediately or up to 6 hours post-intervention [41, 42, 46]; the short-term effect was defined as when the outcome was measured after a short intervention period, ranging from 10 to 12 weeks post-intervention [47, 48].

On the acute effect, one study measured the outcome 20 min after the supplementation [33], two study outcomes were measured 2 hours after the supplementation[37, 43], one study outcome was measured 3 hours after the supplementation [40], and two study outcomes were measured 6 hours after the supplementation [41, 42]. On short-term effects, one study measured the outcome after 10 weeks of intervention [38], one study was 11 weeks [32], three studies were 12 weeks [34–36], one study was approximately 13 weeks (which is 90 days) [39], and one study was 16 weeks [31]. Upon reviewing the results from these articles, the majority showed that supplementation demonstrated a significant effect on attention up to 6 h (i.e. the acute effect) post supplementation [33]. Similarly, majority of the studies also showed that supplementation had a significant short-term effect on memory and attention periods of across studies [31–36, 36, 38–42].

### Cognitive measurements and outcomes

The outcome measures in all the studies are summarized in Table 2, and the quatitative analysis is summarized in Tables 3 and 4.There was a total of 24 types of cognitive tests in the 13 articles. The domains of cognitive tests in these studies included attention and memory [31–43].

**Table 2:** Description of cognitive measurements

Author	Cognitive function domain	Cognitive test
(Carrillo et al., 2021) [31]	Selective attention	Stroop Test
	Working memory	Reynolds Intellectual Screening Test (RIST)
(Chung et al., 2019) [32]	Selective attention	Frankfurt Attention Inventory (FAIR)
	Sustained attention and selective attention	Auditory Continuous Performance Test
	Working memory	Digit Span Forward
	Working memory	Digit Span Backwards
(Rosli et al., 2021) [38]	Memory recall	Digit Forward and Digit Backwards Span Task
	Divided attention	Comprehensive Trail Making Test (CTMT)
(Thukham-Mee et al., 2020) [40]	Selective attention	Stroop Test
	Working memory	Computerized Battery Test
(Barfoot et al., 2019) [42]	Delayed memory	Rey Auditory Verbal Learning Test (RAVLT)
	Selective attention	Modified Attention Network Task (MANT)
(Dodd et al., 2019) [43]	Short-term memory	Global Cognitive Function
	Selective attention	Stroop Test
(Haskell-Ramsay et al., 2017) [33]	Attention and memory recall	Computerized Mental Performance Assessment System (COMPASS)
(Kent et al., 2015) [34]	Long-term memory	Rey Auditory Verbal Learning Test (RAVLT)
	Semantic memory	Boston Naming Test
	Working memory	Digit Span Backwards Task
	Working memory	Self-Ordered Pointing Task
	Semantic memory	Boston Naming Test
(Krikorian et al., 2009) [35]	Memory recall	California Verbal Learning Test (CVLT)
	Spatial memory	Spatial Paired Associate Learning Test
(Lamport, Lawton, et al., 2016) [36]	Short-term and long-term memory	Visual Verbal Learning Test (VVLT)
	Short-term and long-term visual-spatial memory	Visual-Spatial Learning Test (VSLT)
(Lamport, Pal, et al., 2016) [37]	Selective attention	Stroop Test
	Sustain attention and inhibition	Go-Nogo Task
	Short-term memory	Letter Memory Test Logical Memory Immediate Recall Immediate Word Recall
	Long-term memory	Delayed Word Recall
	Spatial memory	Spatial Delayed Recall Test
(Miller et al., 2017) [39]	Memory	California Verbal Leaning Test
(Whyte et al., 2015) [41]	Short-term memory	Modified Rey’s Auditory Verbal Learning (RAVLT)

**Table 3:** Description of the effect of intervention on attention

Cognitive function	Author	Cognitive test name	Test score mean ± SD changes		*p* Value of interaction effect
			**Intervention**	**Placebo/control**	
**Selective** **attention**	Carrillo et al., 2021 [31]	Stroop Test	4.67 ± NR	1.56 ± NR	< 0.05
	Dodd et al., 2019 [43]		0.22 ± NR	-2.63 ± NR	0.81
	Lamport et al., 2017		-24 ± NR	-28 ± NR	0.71
	Thukham-mee et al., 2020 [40]		2.72 ± NR	4.27 ± NR	0.501
	Chung et al., 2019 [32]	FAIR	31.08 ± 2.6	21.22 ± 9.9	< 0.05
	Barfoot et al., 2019 [42]	MANT: Accuracy	6.01 ± NR	7.74 ± NR	0.68
		MANT: Reaction time	-11.09 ± NR	-4.08 ± NR	0.21
**Divided attention**	Rosli et al., 2021 [38]	CTMT: Trail 1	4.1 ± NR	-0.1 ± NR	0.05
		CTMT: Trail 2	-1.9 ± NR	5 ± NR	0.62
		CTMT: Trail 3	1.1 ± NR	1 ± NR	0.45
		CTMT: Trail 4	-3.2 ± NR	4.2 ± NR	0.29
		CTMT: Trail 5	2.4 ± NR	-1.8 ± NR	0.37
		CTMT: Composite Index	4.8 ± NR	-2.6 ± NR	0.08
**Sustained attention** **and selective attention**	Chung et al., 2019 [32]	Auditory Continuous Performance Test	5.16 ± 3.54	9.28 ± 4.39	0.05
**Sustained attention** **and inhibition**	Lamport et al., 2017	Go-No-Go Task	5 ± NR	-7 ± NR	0.86
**Sustained attention** **and vigilance**	Haskell-Ramsay et al., 2017 [33]	COMPASS: Accuracy	0.22 ± NR	0.12 ± NR	0.48
		COMPASS: Reaction Time	-0.21 ± 0.15	0.16 ± 0.15	0.041

Note. *FAIR* Frankfurt Attention Inventory, *TMT* Trail Making Test, *CTMT* Comprehensive Trail Making Test, *MANT* Modified Attention Network Task, *COMPASS* Computerized Mental Performance Assessment System

**Table 4:** Description of the effect of intervention on memory

	**Author**	**Cognitive test name**	**Test score mean ± SD changes**		***P* value of interaction**
			**Intervention**	**Placebo/control**	
**Composite memory recall**	Haskell-Ramsay et al., 2017 [33]	COMPASS—Accuracy	-0.23 ± NR	-0.53 ± NR	0.21
		COMPASS—Reaction time	-0.31 ± NR	-0.2 ± NR	0.84
	Krikorian et al., 2009 [35]	CVLT	1.2 ± NR	-0.4 ± NR	0.1
**Short-term memory recall**	Rosli et al., 2021 [38]	RAVLT	5.00 ± NR	-11.5 ± NR	0.06
	Barfoot et al., 2019 [42]		-0.53 ± NR	-1.23 ± NR	0.04
	Kent et al., 2015 [34]		3.9 ± 0.88	-1.8 ± 3.85	0.014
	Whyte et al., 2015 [41]	RAVLT	-0.8 ± NR	-0.8 ± NR	< 0.001
			-2.5 ± NR	-3.5 ± NR	< 0.001
	Lamport et al., 2016 [36]	VVLT	2.2 ± NR	1.6 ± NR	< 0.05
		VVLT with Retroactive Interference	3.2 ± NR	-4.00 ± NR	< 0.05
		VVLT with Retroactive Interference	14.3 ± NR	4.6 ± NR	< 0.05
	Lamport et al., 2017	Letter Memory	0.5 ± NR	0.1 ± NR	0.89
		Logical Memory Immediate Recall	-2.2 ± NR	-2.1 ± NR	0.97
		Immediate Word Recall	0 ± NR	-0.3 ± NR	0.11
	Dodd et al., 2019 [43]	Global Cognitive Function	7.59 ± 1.38	7.41 ± 1.38	0.19
	Miller et al., 2017 [39]	Digit Span Task	NR	NR	Not significant
**Long-term memory recall**	Rosli et al., 2021 [38]	RAVLT	4.4 ± NR	3.3 ± NR	0.061
	Barfoot et al., 2019 [42]		-0.87 ± NR	-1.77 ± NR	0.164
	Kent et al., 2015 [34]		1.6 ± 0.4	0.2 ± 0.4	0.005
	Miller et al., 2017 [39]	CVLT-II	0.1 ± NR	-0.2 ± NR	Not significant
	Lamport et al., 2016 [37]	VVLT	0.6 ± NR	1 ± NR	Not significant
	Lamport et al., 2017	Delayed Word Recall	-2 ± NR	-0.7 ± NR	0.15
	Dodd et al., 2019 [43]	Global Cognitive Function	5.51 ± 1.98	5.92 ± 2.04	0.95
**Immediate recognition**	Dodd et al., 2019 [43]	Global Cognitive Function	26.65 ± 1.8	26.15 ± 1.77	0.32
**Delayed recognition**	Dodd et al., 2019 [43]	Global Cognitive Function	21.51 ± 3.21	22.89 ± 3.03	0.067
**Working memory**	Carrillo et al., 2021 [31]	RIST	15.27 ± NR	2.28 ± NR	< 0.05
	Rosli et al., 2021 [38]	Digit Span Forward	0 ± NR	0 ± NR	0.48
	Chung et al., 2019 [32]		12.33 ± 2.60	-3.19 ± 1.98	< 0.05
	Rosli et al., 2021 [38]	Digit Span Backward	0.3 ± NR	-0.1 ± NR	0.34
	Kent et al., 2015 [34]		0.4 ± NR	0.5 ± NR	NR
	Chung et al., 2019 [32]		4.42 ± 0.15	9.03 ± 6.56	< 0.05
	Thukham-mee et al., 2020 [40]	Digit Updating	-9.23 ± NR	-3.79 ± NR	0.61
		CBT ((Picture Updating-0 Back)	1.74 ± NR	-0.19 ± NR	0.98
		CBT (Picture Updating-1 Back)	-6.17 ± NR	4.88 ± NR	0.85
		CBT (Picture Updating-2 Back)	4.17 ± NR	9.95 ± NR	0.268
		CBT (Flanker Arrow)	-5 ± NR	-3.97 ± NR	0.56
		CBT (Left Right)	0.75 ± NR	0.13 ± NR	0.16
		CBT (Up Down)	-4.35 ± NR	-2.62 ± NR	0.65
		CBT (Switch-Up Down-Left–Right)	0.75 ± NR	2.47 ± NR	0.78
		CBT (Odd Even)	-4.02 ± NR	-0.72 ± NR	0.991
		CBT (Vowel Consonant)	-5.30 ± NR	5.34 ± NR	0.35
		CBT (Switch Letter Number	-0.98 ± NR	1.4 ± NR	0.51
	Kent et al., 2015 [34]	Self-Ordered Pointing Task	-1.00 ± NR	-0.6 ± NR	NR
	Rosli et al., 2021 [38]	Digit Span Forward	0 ± NR	0 ± NR	0.48
	Chung et al., 2019 [32]		12.33 ± 2.60	-3.19 ± 1.98	< 0.05
	Rosli et al., 2021 [38]	Digit Span Backward	0.3 ± NR	-0.1 ± NR	0.34
	Kent et al., 2015 [34]		0.4 ± NR	0.5 ± NR	NR
	Chung et al., 2019 (32		4.42 ± 0.15	9.03 ± 6.56	< 0.05
	Thukham-mee et al., 2020 [40]	Digit Updating	-9.23 ± NR	-3.79 ± NR	0.61
		CBT ((Picture Updating-0 Back)	1.74 ± NR	-0.19 ± NR	0.98
		CBT (Picture Updating-1 Back)	-6.17 ± NR	4.88 ± NR	0.85
		CBT (Picture Updating-2 Back)	4.17 ± NR	9.95 ± NR	0.268
		CBT (Flanker Arrow)	-5 ± NR	-3.97 ± NR	0.56
		CBT (Left Right)	0.75 ± NR	0.13 ± NR	0.16
		CBT (Up Down)	-4.35 ± NR	-2.62 ± NR	0.65
		CBT (Switch-Up Down-Left–Right)	0.75 ± NR	2.47 ± NR	0.78
		CBT (Odd Even)	-4.02 ± NR	-0.72 ± NR	0.991
		CBT (Vowel Consonant)	-5.30 ± NR	5.34 ± NR	0.35
		CBT (Switch Letter Number	-0.98 ± NR	1.4 ± NR	0.51
	Kent et al., 2015 [34]	Self-Ordered Pointing Task	-1.00 ± NR	-0.6 ± NR	NR
**Spatial memory**	Krikorian et al.,2009 [35]	Spatial Paired Associate Learning Test	1.7 ± NR	-0.4 ± NR	0.12
	Lamport et al., 2017	Spatial Delayed Recall Test	1.8 ± NR	0.9 ± NR	0.68
	Lamport et al., 2016 [36]	VSLT	2.8 ± NR	0.6 ± NR	< 0.05
	Lamport et al., 2016 [37]		0.8 ± NR	0.1 ± NR	< 0.05
	Lamport et al., 2017	Logical Memory Delayed Recall	-1.2 ± NR	-2 ± NR	0.48
**Semantic memory**	Kent et al., 2015 [34]	Boston Naming Test	0.6 ± NR	-1.2 ± NR	NR

Note. *RIST* Reynolds Intellectual Screening Test, *CBT* Computerized Battery Test, *CVLT* California Verbal Learning Test, *COMPASS* Computerized Mental Performance Assessment System, *RAVLT* Rey Auditory Verbal Learning, *VVLT*Visual Verbal Learning Test, *VVLT* Visual Verbal Learning Test, *VSLT* Visual Spatial Learning Test

### Attention

In this review, eleven studies measured attention using seven different cognitive tests. Each test assessed a different aspect of attention.

The selective attention tests, including Stroop Test, Frankfurt Attention Inventory (FAIR), and Modified Attention Network Task (MANT), were employed. The Stroop Test was used by multiple studies and measured by Carrillo et al. [31] after 16 weeks of dehydrated fruits and vegetables capsules supplementation, Dodd et al. [43] after 2 hours of freeze-dried blueberry supplementation, Thukham-Mee [40] after 3 hours of mulberry powder supplementation, and Lamport et al. [37] after 2 hours of fruit juice supplementation. Stroop Test assesses the individual’s ability to focus on a relevant stimulus (colour of the words shown) while ignoring irrelevant information (meaning of the word) [49]. The results only showed significant improvement in selective attention with short-term supplementation, and this improvement was not observed in the immediate measurement of attention after the supplementation [31]. However, the result has to be carefully interpreted as these studies have different supplementation which may exhibit different outcomes. Additionally, a recent study has questioned the reliability of the Stroop Test in studying attention [50]; thus, careful consideration must be taken when choosing the selective attention test.

FAIR was used by Chung et al. [32], and this test shares a similar pattern with the Stroop Test, but the FAIR test is quicker to do, and it also involves identifying the stimulus while ignoring the distractors [51]. Chung et al. [32] measured the outcome after 11 weeks of Lycium supplementation, and the results showed significant improvement.

MANT is used by Barfoot et al. [42] where participants were required to respond to the relevant stimuli (the direction of a single arrow appearing opposite to many other arrows) and ignore the distractors. In addition, MANT involves not only selective attention, but also orientation, alertness, and executive control [52]. The outcome was measured 2–6 hours after freeze-dried blueberry supplementation, and the results did not show any significant improvement.

Rosli et al. [38] utilized the Comprehensive Trail Making Test (CTMT), a measure of divided attention [53]. Divided attention is the cognitive ability to focus on multiple tasks simultaneously. CTMT is derived from Trail Making Test (TMT) and consists of two additional tasks compared with TMT to make up a total of five tasks. CTMT overcomes the shortcomings of TMT while increasing the difficulty and complexity of the tasks [53]. The task requires participants to shift between numbers and letters simultaneously. However, it is noteworthy that TMT lacks specificity in discriminating executive function from working memory [54–56]. Rosli et al. [38] measured the divided attention after 10 weeks of juice extract supplementation, and the result showed significant improvement.

Attention is a complex process, and attention tests such as Auditory Continuous Performance Test, and Go-Nogo Task used in the reviewed studies assess multiple of attention.

Chung et al. [32] utilized Auditory Continuous Performance Test, measuring sustained attention and selective attention over auditory stimuli [32, 57]. This test examines the ability to maintain focus on auditory stimulus over a long period of time, without being distracted. The outcome was measured after 11 weeks of Lycium capsule supplementation, and the result showed significant improvement.

Lamport et al. [37] utilized the Go-Nogo Task to measure the sustained attention and inhibition where the task required participants to maintain their focus and respond to relevant stimuli (Go) and inhibit the irrelevant stimuli (Nogo) [37]. The cognitive function was measured after 2 hours of fruit juice supplementation, and the result did not show any significant improvement.

Haskell-Ramsay et al. [33] used Computerized Mental Performance Assessment System (COMPASS). COMPASS is a computerized test with a cluster of cognitive tests which includes assessing memory and attention. The attention tests included the attention aspect of vigilance and sustained attention. Participants are required to maintain their focus for an extended period of time and respond to the stimuli when the stimuli appear. The attention tests were measured after 20 min of grape juice supplementation, and the results showed a significant improvement in the reaction time, but not in accuracy.

Based on the different cognitive tests measuring attention in this review, we observed a significant improvement in selective attention, sustained attention, and divided attention with supplementation of fruit and vegetable intake, particularly in the short-term studies ranging from 11 to 16 weeks [31, 32, 38]. However, when examining the acute effects, where measurements were conducted 2 to 3 hours after the supplementation, no significant improvement was found [37, 40, 43]. In light of these findings, it is essential to be mindful of the timing of the post-supplementation measurement.

### Memory

Different aspects of memory were assessed in the various studies, for example, memory was evaluated as composite memory, short-term memory, long-term memory, working memory, immediate and delay memory, semantic memory, and spatial memory.

Composite memory was measured by the combination of various memory domains, for example, the Computerized Mental Performance Assessment System (COMPASS) and California Verbal Learning Test (CVLT).

Haskell-Ramsay et al. [33] utilized COMPASS which combines episodic memory and working memory to evaluate memory function as whole. Krikorian et al. [35] used CVLT to measure the composite score of short-term and long-term memory recall. CVLT is a measure that is derived from the Auditory Verbal Learning Test, and it includes different cognitive measures such as short-term memory recall and recognition, long-term memory recall and recognition, and learning strategies.

Krikorian [35], who used CVLT to test memory recall, showed a significant improvement after 12 weeks of grape juice supplementation in the supplementation group compared to placebo. However, the study by Haskell-Ramsay et al. [33] did not show any significant improvement after 20 min of freeze-dried blueberry supplementation. Although both Krikorian [35] and Haskell-Ramsay et al. [33] showed contradictory results in composite memory, it could have been because of the difference in length of the supplementation where it was much longer (12 weeks) by Krikorian [35] compared with a much shorter period of supplementation Haskell-Ramsay et al. [33].

Eight studies measured short-term memory recall by using the Rey Auditory Verbal Learning Test (RAVLT), Visual Verbal Learning Test (VVLT), Letter Memory, Logical Memory, Immediate Recall, Global Cognitive Function, and Digit Span Task [34, 36–39, 41–43].

Three studies used the RAVLT [34, 38, 42], and one study used the modified RAVLT [41] to test short-term memory. The results were mixed with the RAVLT test on short-term memory recall with different durations of intervention. Barfoot et al. [42], Kent et al. [34], and Whyte et al. [41] showed a significant improvement in attention when the RAVLT test was administered at 6 hours [41, 42] and 12 weeks [34] of consumption of the supplement. However, the result from Rosli and colleagues (2021), who had administered the RAVLT after 10 weeks of supplementation consumption, did not show any significant improvement in short-term memory.

Lamport et al. [36] utilized VVLT to measure short-term memory recall. VVLT serves as a visual analogue of the RAVLT. The result was a significant improvement of short-term memory recall after 12 weeks of grape juice supplementation.

Lamport et al. [37] utilized multiple memory tests which included Letter Memroy, Logical Memory (which is adapted from Wechsler Memory scale [36] which originally measured verbal episodic memory including immediate recall, delayed recall, and delay recognition [58]). However, the results did not show any significant effect on the any of the memory tests after 2 hours of fruit juice supplementation.

Dodd et al. [43] used a global cognitive function test which consists 14 tasks assessing different cognitive domains (memory, attention, executive function). This global cognitive function test also includes short-term memory. Results did not show any significant improvement after 2 hours of freeze-dried blueberry supplementation.

Miller et al. [39] used the Digit Span Task to measure short-term memory with immediate recall and short-delay recall in older adults. The results showed no significant improvement in short-term memory recall after approximately 13 weeks of blueberry powder supplementation.

Another aspect of memory that was found in this review is long-term memory recall. Long-term memory recall was measured in seven studies with RAVLT, CVLT, VVLT, Delayed Word Recall, and Global Cognitive Function [34, 36–39, 42, 43]. Out of these seven studies, only Kent et al. [34] in a 12-week intervention study showed a significant improvement measured by CVLT. Furthermore, Lamport et al. [36] also showed a significant improvement with a 12-week intervention measured by VVLT, while the rest of the studies did not show significant changes.

With reference to memory, there were studies which measured immediate and delay recognition. Immediate and delayed recognition was measured by Dodd et al. [43] using Global Cognitive Function. However, the result was not significant after 2 hours of freeze-dried blueberry supplementation.

Working memory was also evaluated in the studies of this review. Working memory was measured in five studies with different cognitive task such as Digit Span Test [32, 38], Self-ordered Pointing Task, Reynold Intellectual Screening Test (RIST) [31], and Computerized Battery Test designed by Thukham-Mee [40] consisting 10 memory tasks. RIST is a brief screening measure of intelligence [59], but Carrillo [31] used it to evaluate working memory.

The results showed a significant improvement after 16 weeks of intervention using RIST [31], similar to Chung’s et al. [32] study, whose outcome was measured by Digit Span Task after 11 weeks of intervention. However, Rosli [38], who also used the Digit Span Task with 10 weeks of intervention, did not show any significant improvement after the intervention. These two studies presented contradictory results due probably to different supplements consisting of different micronutrients and different durations. Chung et al. [32] used Lycium, which contained 3.41 mg/g betaine, for 11 weeks, while Rosli [38] used a mixture of pomegranate with guava and roselle extract, which contained 609 mg/100 ml phenolic, for 10 weeks.

Furthermore, Thukham-Mee et al. [40] measured working memory outcomes after 3 hours of supplementation using a computerized battery test, and Kent et al. [34], who conducted a 12-week supplementation and measured the outcome using the Self-ordered Pointing Task, did not report any significant differences in working memory.

Another aspect of memory is spatial memory which refers to ability to remember the location, physical arrangement of objects, and features of the environment [60]. Spatial memory was measured in five studies by using the Visual Spatial Learning Test (VSLT), Visual Spatial Learning Test (VSLT), Spatial Paired Associate Learning Test, and Spatial Delayed Recall Test [35–37]. Among all these different measurements, only Lamport et al. [36] showed a significant improvement in spatial memory with 12 weeks of grape juice supplementation using the VSLT.

The last aspect of the memory in this review is sematic memory which refers to general knowledge about the world [61]. Semantic memory was measured by Kent et al. [34] using the Boston Naming Test. However, the result was not significant after 12 weeks of cherry juice supplementation.

## Discussion

This systematic review summarizes 13 studies that examined the effect of fruit and vegetable consumption on cognition. In general, out of 13 studies, 55.3% of longitudinal studies showed a significant improvement in memory and attention for the supplementation group compared with the placebo group. However, only 8.8% studies on acute effects showed significant improvement. The divergent outcome on effect between longitudinal and acute supplementation prompts the need for exploration into the temporal dynamics of the effects of fruit and vegetable intake on cognitive function. Previous research using polyphenols, derived from fruits and vegetables, showed accumulation in the hippocampus [62], an important region for memory functioning [63] and for increasing neurogenesis [64]. In addition, this result is aligned to a 25-year prospective study which showed that vegetable intake is associated with better cognitive performance [65]. Thus, a longer duration of supplementation may be needed to modulate brain function. Based on our reviews, a few studies using polyphenols also reported significant improvement in memory [31, 36].

Different supplements were used in these 13 studies. Four out of these 13 studies used blueberries as an intervention to test the effect on attention and memory [39, 41–43]. Blueberries were dispensed in the form of freeze-dried blueberries [42, 43] and blueberry powder [39, 41]. Immediate memory recall may be improved with longer-term consumption of blueberries. Based on our review, consumption of blueberry powder for a longer duration of 12 weeks showed significant improvement in immediate memory recall measured by CVLT for older adults [39] compared with other studies that measured the effect after 2 hours of consumption [43]. However, studies on children’s immediate memory recall 6 hours after consumption of blueberry powder or fresh blueberries showed a significant improvement as measured by the RVLT [41, 42]. This suggests that a longer duration of consumption is needed for a significant improvement in immediate memory for older adults, while in children, short-term consumption seems to work positively. These results raised concerns of the underlying physiological factor of different age groups on cognitive function. Considering the physiological changes associated with aging, older adults experience reduction in digestive enzymes [66], potentially impacting the breakdown of molecular bonds and absorption of nutrients [67]. In general, 65% of polyphenols are released in the stomach, and 10% are released in the intestine [68]. However, bioavailability of polyphenols by older adults may be influenced by the efficiency of the digestive system. Consequently, smaller amounts of micronutrients are absorbed by older adults compared with children, perhaps explaining the improved memory recall with a longer period of supplementation as it is needed before the nutrients can show an effect. In addition, this finding aligns with the concept of STAC, suggesting that compensatory intervention such as a change of dietary habits can play a crucial role in mitigating cognitive decline associated with aging.

Another supplement used by many researchers was grapes [33, 35, 36]. Grape juice is beneficial for memory with the consumption of 12 weeks based on measurements of CVLT and VSLT [35, 36]. Furthermore, a higher dose of grape juice (621 ml) per day for 12 weeks is needed in the older adult population to significantly improve memory [35] than in the younger adult population, where only 350 ml was needed to show a significant change in memory [36]. This again suggests and supports that either higher doses of supplementation or longer duration (which effectively results in higher total amount of supplementation) are needed in older adults for it to have a beneficial effect.

 Both blueberries and grapes have a positive effect on cognitive function, which could be explained by biological mechanisms. Blueberries and grapes have a high polyphenol content, which serves as an antioxidant [69], slowing oxidative stress [70, 71] and thus reducing oxidative damage [8, 9, 72]. In addition, pterostilbene (PTS), one of the polyphenols, found in blueberries and grapes has been shown to have various other effects, such as antioxidant, antiinflammation, and anticancer [73, 74]. With its benefits, it may serve as a protective factor against cognitive deterioration and neurodegenerative diseases such as Alzheimer’s disease and other dementias.

The nutritional value of fruits and vegetables can be affected by many factors, including the method of processing and storage. For example, microbial contamination of goji berries varies depending on the country of cultivation and its preservation methods [75]. To overcome and preserve the degradation of nutrients, studies using capsule supplementation have been done. Researchers extracted dehydrated fruit and vegetable [31] or dried Lycium Chinese fruits [32] and repackaged them into capsule form. Both studies which administered the supplement in capsule form, showed a significant improvement in memory and attention. The encapsulation of supplement protects the bioactive compounds from oxidation and degradation due to light exposure [76] or processing temperature [77]. However, it also requires professional skills to reproduce the capsules, and this is done at a higher production cost.

### Strengths and limitations

This review summarizes the effect of consumption of fruit and vegetable intake on memory and attention from 13 articles. The review focuses on randomized control trials that minimized other confounding variables, and it indicates that fruit and vegetable consumption based on several experimental studies have positive effects on cognitive functions.

However, there are several limitations to this review. A small number of available studies with different forms of fruit and vegetable intake with different micronutrient contents and different outcome measures have made it difficult to draw a definitive conclusion on the dose/quantity/duration that are needed to achieve a significant improvement in memory and attention. In addition, 26 different cognitive tests were used to measure cognitive function in these studies. It is difficult to draw conclusions on the sensitivity of different cognitive tests due to a lack of comparison using the same test.

## Conclusion

The studies included in this systematic review highlighted the effects of fruit and vegetable intake on memory and attention. This systematic review showed that fruit and vegetable intake consumption significantly improved both attention and memory when the consumption lasted for 10 to 12 weeks. There are different forms of fruit consumption, such as fresh juice, powder extract, or capsule. Children who consumed blueberries showed improvement on the immediate recall test. In addition, older adults have better memory recall with a longer consumption of 12 weeks of blueberry powder consumption by using the CLVLT, and they need a higher dosage of consumption. In conclusion, this systematic review showed that fruit and vegetable intake consumption tended to improve memory and attention. Hence, awareness of the benefit of fruit and vegetable intake consumption is important and should be encouraged to maintain cognitive health.

### Supplementary Information

Supplementary file1.

## Data Availability

The data supporting this systematic review are from previously reported studies and datasets, which have been cited. The processed data are available from the corresponding author upon request.
