# Supplementary material for: Effects of fruit and vegetable intake on memory and attention: a systematic review of randomized controlled trials
Source: Syst Rev. 2024 Jun 7;13:151. doi: 10.1186/s13643-024-02547-8 (PMC11157787; doi:10.1186/s13643-024-02547-8)

Appendix 1: Search Terms of Fruits and Vegetable and different types of attention and memory in RCT design

Search	Search terms	Ebscohost (Medline, APA PsychArticles, APA PsycInfo, CINAHL)	ProQuest Central	PubMed	EMBASE	Web of Science
#1	Fruit OR vegetable OR plant capsule	173,074	1,806,661	95,540	107,932	336,901
#2	selective attention OR divided attention OR sustained attention OR attention	832,035	3,214,882	462,217	576,338	985,289
#3	memory OR sensory memory OR Short-term memory OR long-term memory OR working memory OR episodic memory OR semantic memory OR procedural memory OR autobiographical memory OR iconic memory OR echoic memory OR semantic memory OR declarative memory	568,449	2,094,286	276,572	346,180	652,121
#4	Intervention OR randomized OR RCT or placebo* OR clinical trial	3,135,912	2,188,065	1,359,100	1,970,924	2,154,737
#5	#1 AND #2 AND #3 AND #4	18	15	9	14	14

Appendix 1(a): Screenshot search of Ebscohost

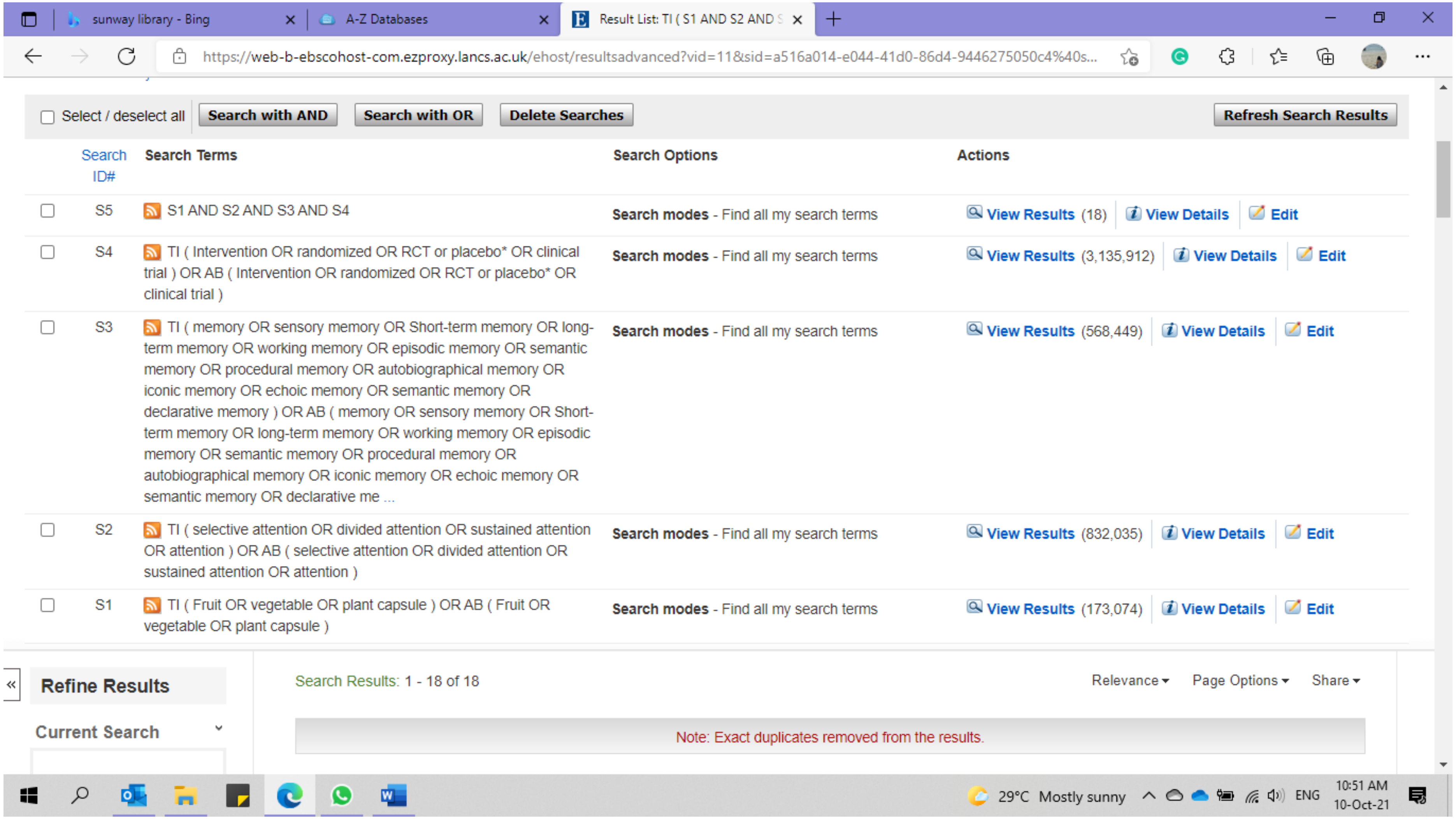

Appendix 1 (b): Screenshot search for Web of Science

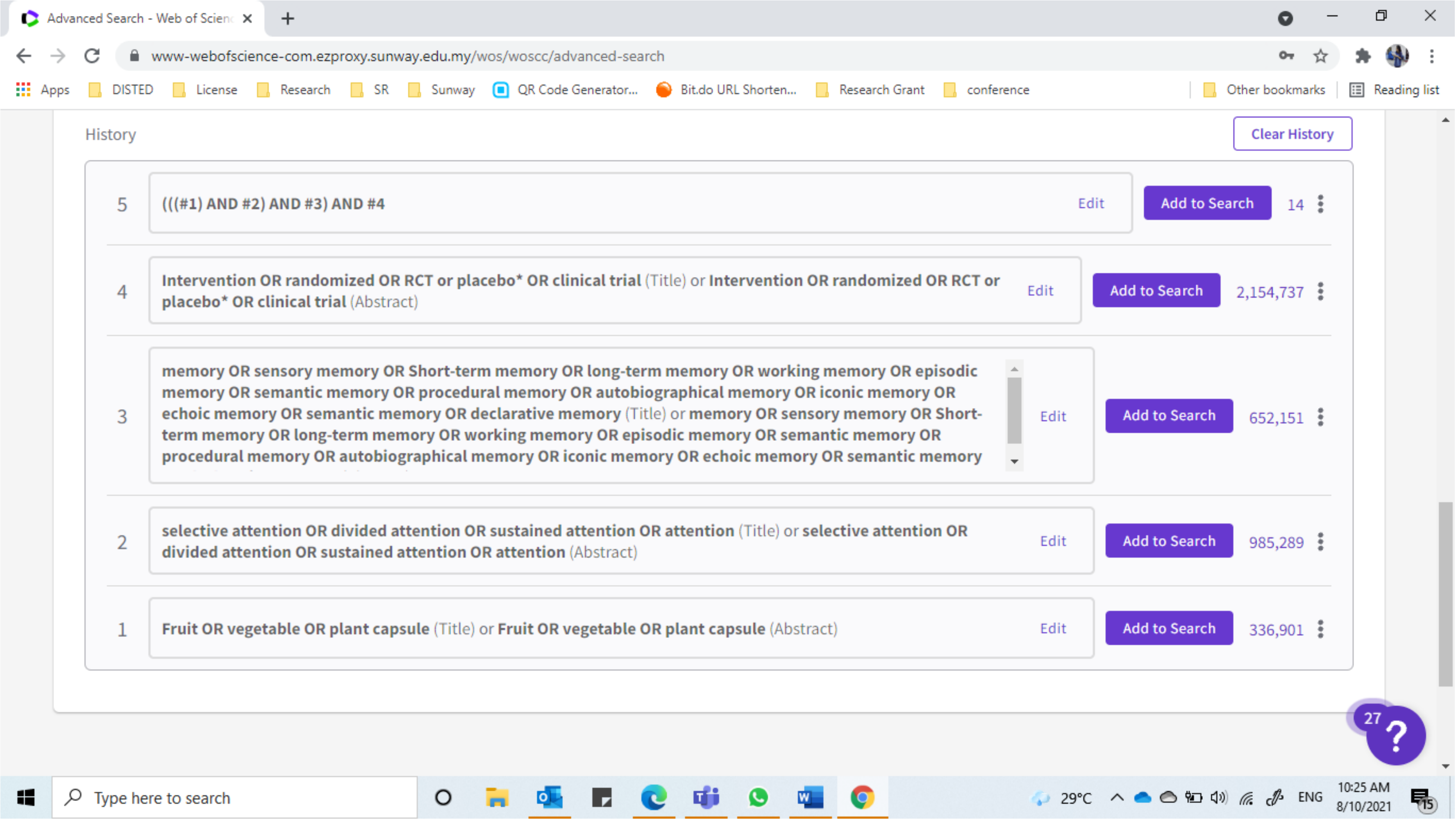

Appendix 1(c): screenshot search for Proquest

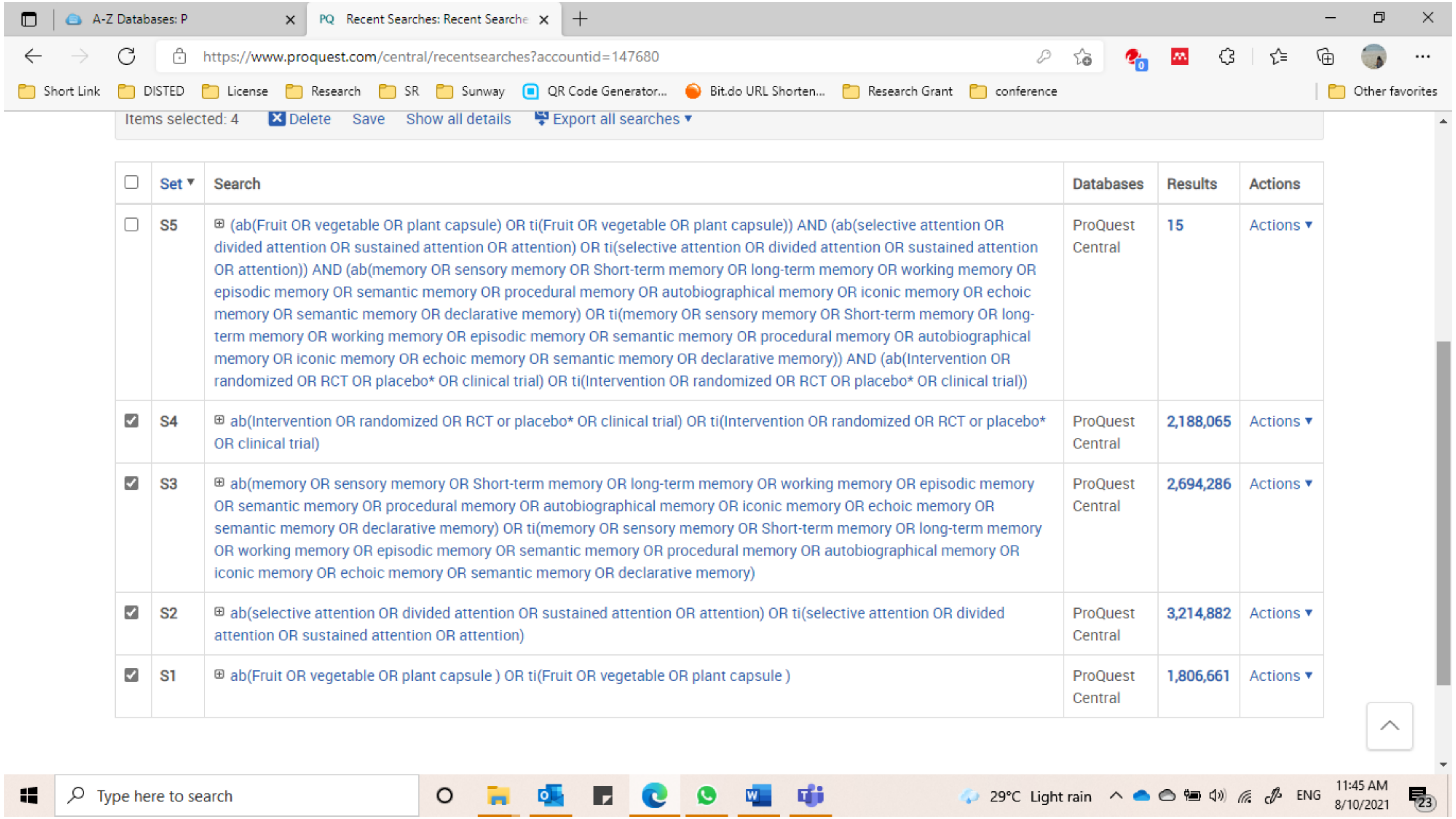

Set	Search	Databases	Results	Actions
S5	(ab(Fruit OR vegetable OR plant capsule) OR ti(Fruit OR vegetable OR plant capsule)) AND (ab(selective attention OR divided attention OR sustained attention OR attention) OR ti(selective attention OR divided attention OR sustained attention OR attention)) AND (ab(memory OR sensory memory OR Short-term memory OR long-term memory OR working memory OR episodic memory OR semantic memory OR procedural memory OR autobiographical memory OR iconic memory OR echoic memory OR semantic memory OR declarative memory) OR ti(memory OR sensory memory OR Short-term memory OR long-term memory OR working memory OR episodic memory OR semantic memory OR procedural memory OR autobiographical memory OR iconic memory OR echoic memory OR semantic memory OR declarative memory)) AND (ab(Intervention OR randomized OR RCT OR placebo* OR clinical trial) OR ti(Intervention OR randomized OR RCT OR placebo* OR clinical trial))	ProQuest Central	15	Actions ▾
S4	ab(Intervention OR randomized OR RCT or placebo* OR clinical trial) OR ti(Intervention OR randomized OR RCT or placebo* OR clinical trial)	ProQuest Central	2,188,065	Actions ▾
S3	ab(memory OR sensory memory OR Short-term memory OR long-term memory OR working memory OR episodic memory OR semantic memory OR procedural memory OR autobiographical memory OR iconic memory OR echoic memory OR semantic memory OR declarative memory) OR ti(memory OR sensory memory OR Short-term memory OR long-term memory OR working memory OR episodic memory OR semantic memory OR procedural memory OR autobiographical memory OR iconic memory OR echoic memory OR semantic memory OR declarative memory)	ProQuest Central	2,694,286	Actions ▾
S2	ab(selective attention OR divided attention OR sustained attention OR attention) OR ti(selective attention OR divided attention OR sustained attention OR attention)	ProQuest Central	3,214,882	Actions ▾
S1	ab(Fruit OR vegetable OR plant capsule) OR ti(Fruit OR vegetable OR plant capsule)	ProQuest Central	1,806,661	Actions ▾

Appendix 1(d): Screenshot search for Pubmed

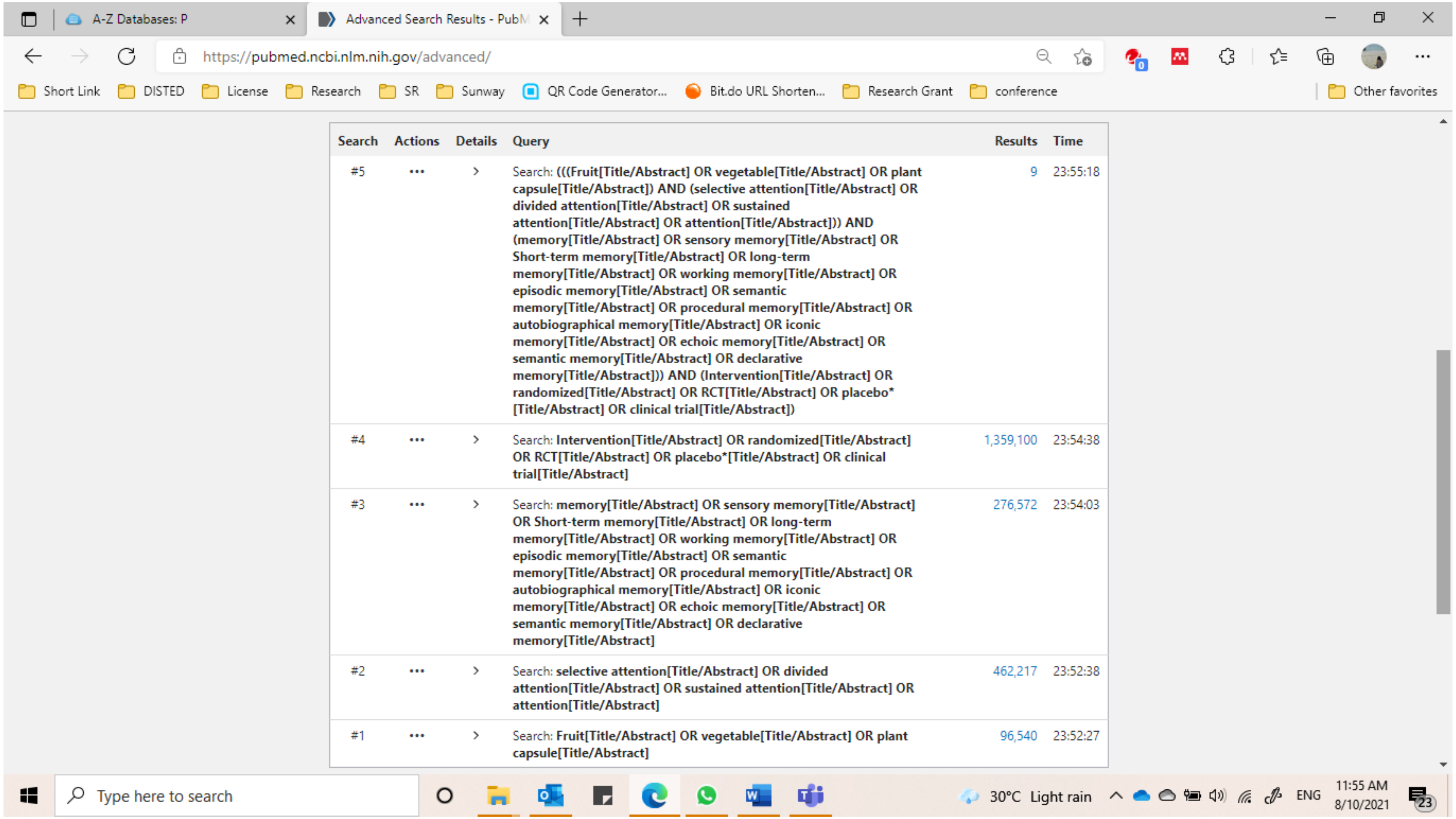

Search	Actions	Details	Query	Results	Time
#5	•••	>	Search: (((Fruit[Title/Abstract] OR vegetable[Title/Abstract] OR plant capsule[Title/Abstract]) AND (selective attention[Title/Abstract] OR divided attention[Title/Abstract] OR sustained attention[Title/Abstract] OR attention[Title/Abstract])) AND (memory[Title/Abstract] OR sensory memory[Title/Abstract] OR Short-term memory[Title/Abstract] OR long-term memory[Title/Abstract] OR working memory[Title/Abstract] OR episodic memory[Title/Abstract] OR semantic memory[Title/Abstract] OR procedural memory[Title/Abstract] OR autobiographical memory[Title/Abstract] OR iconic memory[Title/Abstract] OR echoic memory[Title/Abstract] OR semantic memory[Title/Abstract] OR declarative memory[Title/Abstract])) AND (Intervention[Title/Abstract] OR randomized[Title/Abstract] OR RCT[Title/Abstract] OR placebo*[Title/Abstract] OR clinical trial[Title/Abstract])	9	23:55:18
#4	•••	>	Search: Intervention[Title/Abstract] OR randomized[Title/Abstract] OR RCT[Title/Abstract] OR placebo*[Title/Abstract] OR clinical trial[Title/Abstract]	1,359,100	23:54:38
#3	•••	>	Search: memory[Title/Abstract] OR sensory memory[Title/Abstract] OR Short-term memory[Title/Abstract] OR long-term memory[Title/Abstract] OR working memory[Title/Abstract] OR episodic memory[Title/Abstract] OR semantic memory[Title/Abstract] OR procedural memory[Title/Abstract] OR autobiographical memory[Title/Abstract] OR iconic memory[Title/Abstract] OR echoic memory[Title/Abstract] OR semantic memory[Title/Abstract] OR declarative memory[Title/Abstract]	276,572	23:54:03
#2	•••	>	Search: selective attention[Title/Abstract] OR divided attention[Title/Abstract] OR sustained attention[Title/Abstract] OR attention[Title/Abstract]	462,217	23:52:38
#1	•••	>	Search: Fruit[Title/Abstract] OR vegetable[Title/Abstract] OR plant capsule[Title/Abstract]	96,540	23:52:27

Appendix 1 (e): Screenshot search for Embase

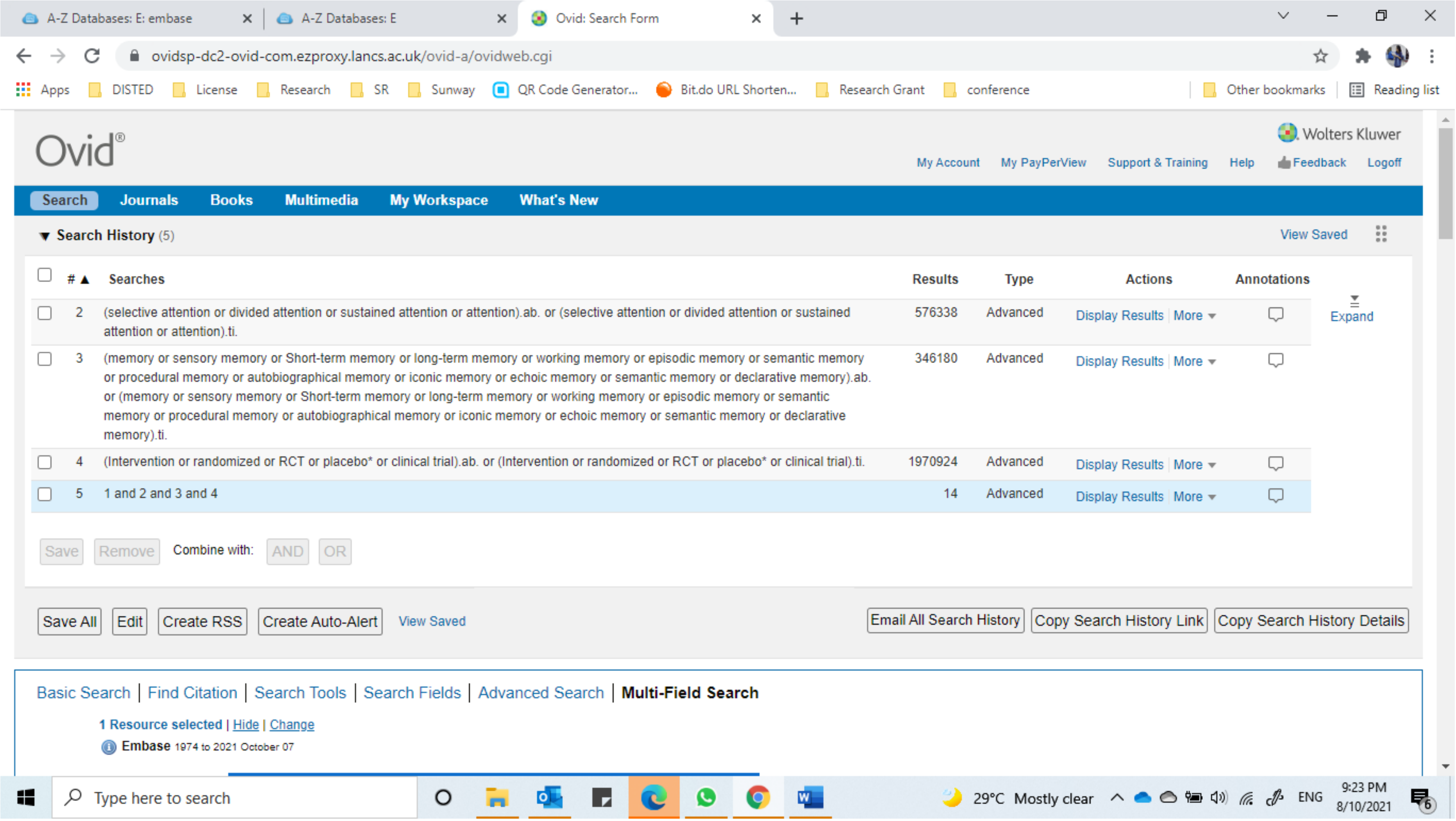

Supplement: Supplementary file 1 — Supplementary file1. [file 13643_2024_2547_MOESM1_ESM.pdf]
